# A novel tissue specific alternative splicing variant mitigates phenotypes in *Ets2* frame-shift mutant models

**DOI:** 10.1038/s41598-021-87751-5

**Published:** 2021-04-15

**Authors:** Yuki Kishimoto, Iori Nishiura, Wataru Hirata, Shunsuke Yuri, Nami Yamamoto, Masahito Ikawa, Ayako Isotani

**Affiliations:** 1 Division of Biological Science, Graduate School of Science and Technology, Nara Institute of Science and Technology, 8916-5 Takayama-cho, Ikoma, Nara 630-0192 Japan; 2 Research Institute for Microbial Diseases, Osaka University, 3-1 Yamadaoka, Suita, Osaka 565-0871 Japan

**Keywords:** Developmental biology, Biological techniques, Biological models, Gene expression analysis, Genetics, Mutation, RNA splicing

## Abstract

E26 avian leukemia oncogene 2, 3′ domain (Ets2) has been implicated in various biological processes. An *Ets2* mutant model (*Ets2*^*db1/db1*^), which lacks the DNA-binding domain, was previously reported to exhibit embryonic lethality caused by a trophoblast abnormality. This phenotype could be rescued by tetraploid complementation, resulting in pups with wavy hair and curly whiskers. Here, we generated new *Ets2* mutant models with a frame-shift mutation in exon 8 using the CRISPR/Cas9 method. Homozygous mutants could not be obtained by natural mating as embryonic development stopped before E8.5, as previously reported. When we rescued them by tetraploid complementation, these mice did not exhibit wavy hair or curly whisker phenotypes. Our newly generated mice exhibited exon 8 skipping, which led to in-frame mutant mRNA expression in the skin and thymus but not in E7.5 *Ets2*^*em1/em1*^ embryos. This exon 8-skipped *Ets2* mRNA was translated into protein, suggesting that this Ets2 mutant protein complemented the Ets2 function in the skin. Our data implies that novel splicing variants incidentally generated after genome editing may complicate the phenotypic analysis but may also give insight into the new mechanisms related to biological gene functions.

## Introduction

E26 avian leukemia oncogene 2, 3′ domain (Ets2), a member of the ETS family, is a transcription factor that contains an ETS winged helix-loop-helix DNA-binding domain (ETS domain) that binds to GGA(A/T) DNA sequences. It is conserved in various species, including mice and humans^1–3^. Ets2 has been implicated in various biological contexts, including placentation, hair formation, mammary tumors, inflammatory responses, angiogenesis, and the pulmonary fibrosis^4–8^.

In a previous study, Ets2-deficient mice (*Ets2*^*db1/db1*^ mice), which lack the ETS domain through deletion of exons 9 and 10, were found to exhibit early embryonic lethality due to a trophectoderm abnormality. The tetraploid complementation technique could rescue this placental abnormality, allowing for survival of the offspring^4^, indicating that Ets2 is essential for placental development. *Ets2*^*db1/db1*^ mice created using the tetraploid complementation technique exhibit a variety of phenotypes, such as wavy hair, curly whisker, and a rounded forehead, allowing them to be identified. However, their fertility is normal, and they exhibit no lethal phenotype after birth. Therefore, the *Ets2*^*db1/db1*^ mouse is a useful model for studying treatment methods for placental abnormalities^9^.

The generation of gene-deficient animal models is now commonly performed using CRISPR/Cas9-based genome engineering^10,11^. Model organisms made using this technique can completely mimic the genome mutations found in human diseases, such as indel mutations and substitutions, which were previously difficult to generate using the conventional knockout method. Further, homozygous mutant mice can be obtained efficiently in the founder generation by directly delivering the crRNA/tracrRNA/Cas9 ribonucleoprotein complex into a mouse zygote via electroporation^12^. Unfortunately, if the homozygous mutant exhibits embryonic lethality, it cannot be obtained in this way. However, it is possible to obtain placental-deficient mutant mice, such as Ets2, in the founder generation using the tetraploid complementation method^13^ in combination with genome-edited zygotes or their embryonic stem cells.

Using the above strategy, we established three new *Ets2* mutant mouse lines. Two of those contained a frame-shift deletion in exon 8, which was located before the ETS domain encoded by exons 9 and 10. These genomic mutations were predicted to produce a transcriptional product that would undergo nonsense-mediated mRNA decay (NMD) or, if translated, a protein lacking the ETS domain. The third one had the null mutant allele which lacked all open reading frame regions. We found that some of the phenotypes exhibited by the frame-shift mutant mice differed from the previous study, whose origin was investigated in this work.

## Results

### Generation of new *Ets2* mutant mice by CRISPR/Cas9 system using zygotes

**Figure 1:**
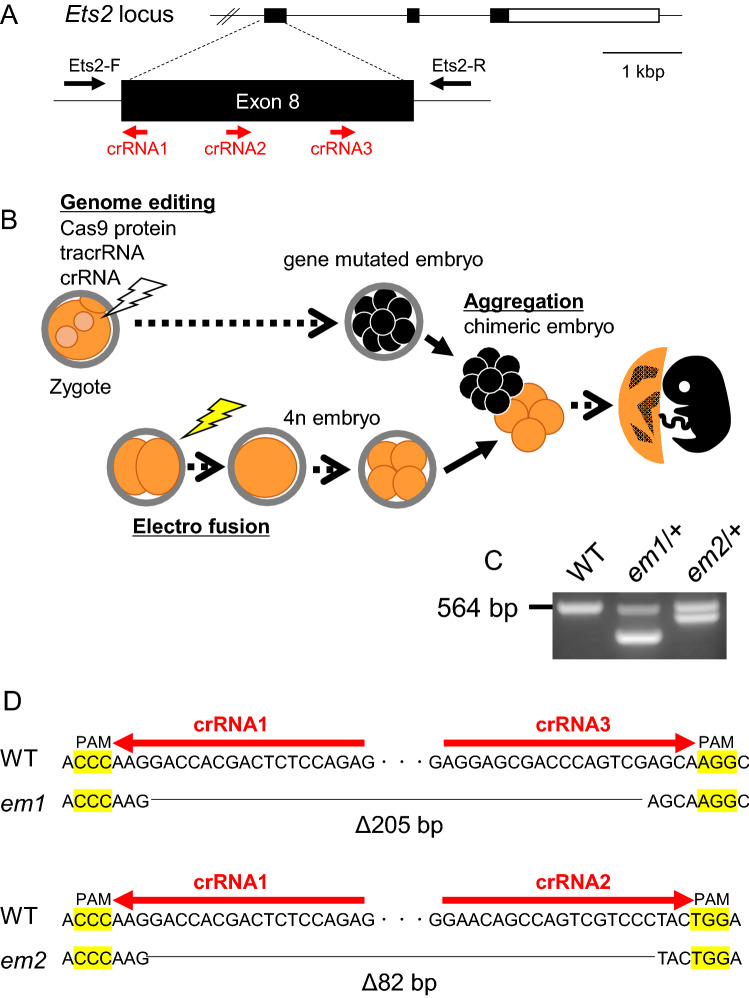
Generation of new *Ets2* mutant models using zygotes. **(A)** Design of crRNA targeting sites in exon 8 of the *Ets2* gene and checking primer positions. **(B)** Strategy for obtaining *Ets2* homozygous mutant l in F0 generation using the electroporation technique and the tetraploid method. **(C)** Genotyping of the two newly generated *Ets2* mutants in F0 generation. Both had a wildtype (WT: 564 bp) allele and a deletion allele (*em1* or *em2*), which were detected as shorter bands than WT. **(D)** DNA sequence of mutant alleles and crRNA targeted sequences. The 205 bp deleted (Δ205 bp) allele was named *em1*, and the 82 bp deleted (Δ82 bp) allele was named *em2*.

On the basis of a previous study^4^, we designed three gRNA targeted to sites in exon 8 that would induce a frame-shift mutation, leading to a deficiency in the ETS domain, encoded by exons 9 and 10 (Fig. 1A). The riboprotein complex, which consisted of three designed crRNAs, tracrRNAs, and Cas9 protein, was electroporated into one-cell stage zygotes, which developed until the eight-cell stage. These genome-edited embryos were used for the tetraploid complementation method in order to obtain homozygous mutant mice in the founder generation (Fig. 1B). However, no homozygous mutant mice were born from the 29 transferred embryos.

Two out of three delivered pups had a heterozygous deletion mutation, which was determined by PCR analysis. One mutation was a 205-bp deletion (hereafter referred to as *em1*), and the other was an 82 bp deletion (hereafter referred to as *em2*) (Fig. 1C,D and full-length gel of Fig. 1C is presented in Supplemental Fig. S1). Expectedly, both had frame-shift mutations.

### Assessment of the development of the newly generated *Ets2* mutant mice

Previous reports indicated that *Ets2*^*db1/db1*^ mice exhibit an embryonic lethal phenotype due to a placental deficiency^4^. Sixteen pups were obtained from three derivations, and as expected, none of the pups included the double mutant alleles (*em1/em2*) (Table 1). Further, we analyzed the developmental ability of *Ets2* mutant mice by performing a test cross using *Ets2*^+*/em1*^ mice and assessed the genotypes of the offspring. No homozygous mutant pups (*Ets2*^*em1/em1*^) were born (wild:hetero:homo = 45:91:0, Table 1).

**Table 1:** Lethality of *Ets2* mutant.

Parents genotype		No. of offspring	Average of litter size	Genotype (*)		
Female	Male			+ */* +	+ */Δ*	*Δ/Δ*
*Ets2*^+*/em1*^	*Ets2*^+*/em2*^	16	5.3	10	6	0
*Ets2*^+*/em1*^	*Ets2*^+*/em1*^	136	6.5 ± 1.7	45	91	0

(*) em1 or em2 allele shown as "Δ".

**Figure 2:**
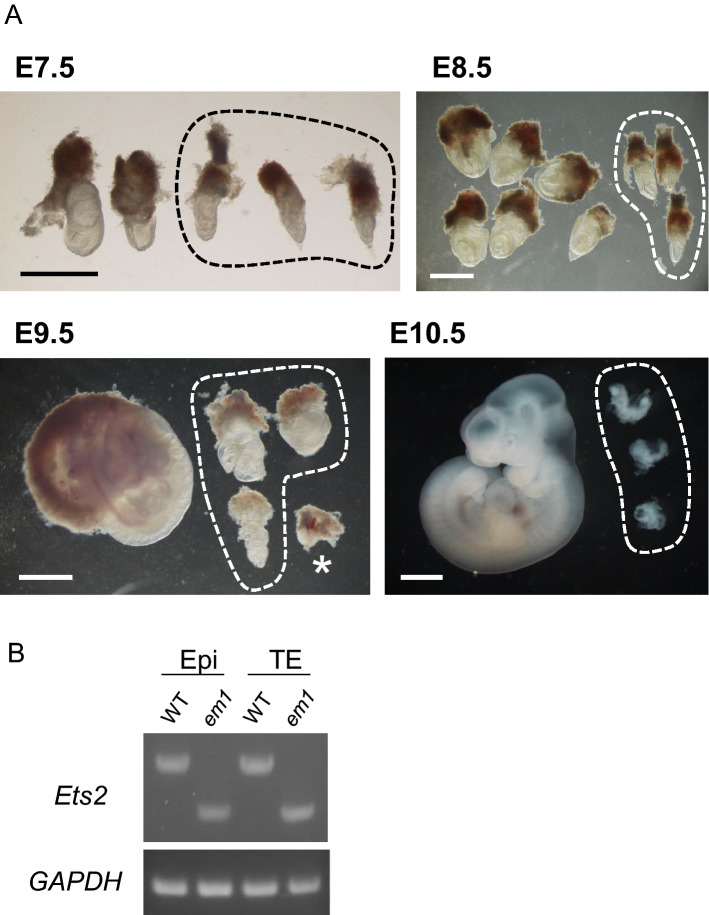
Development of *Ets2*^*em1/em1*^ embryos. **(A)** From E7.5 to E10.5, embryos were observed after crossing *Ets2*^+*/em1*^ females and males. *Ets2*^*em1/em1*^ embryos are indicated as circles in each picture. The regions outside of the circles correspond to *Ets2*^+*/*+^ or *Ets2*^+*/em1*^ embryos. The genotype of the embryo indicated by an asterisk at E9.5 could not be determined. All scale bars indicate 1 mm. **(B)** Gene expressions of E7.5 WT and *Ets2*^*em1/em1*^ (em1) embryos. Embryos were separated into trophectodermal tissue (TE), including ectoplacental corn, and epiblast (Epi), from which RNA and cDNA were prepared. Both em1 bands were shifted to be lower than the WT bands. The DNA sequences of the em1 bands were the same as em1L in Fig. 4B.

A previous study reported that *Ets2*^*db1/db1*^ embryos were degenerated by the placental deficiency around E7.5 and disappeared after E8.5. To investigate whether the *Ets2*^*em1/em1*^ mutant phenocopies the *Ets2*^*db1/db1*^ mutant, we crossed *Ets2*^+*/em1*^ animals and observed embryos at several stages. *Ets2*^*em1/em1*^ embryos at E7.5 were slightly delayed in their developmental stage but clearly progressed in a comparable manner to embryos from *Ets2*^*db1/db1*^ animals. The *Ets2*^*em1/em1*^ embryos had survived at E8.5, but all of them were retarded. By E9.5 and E10.5, some malformed *Ets2*^*em1/em1*^ embryos were present and developed before the turning of the embryo, which usually occurred at approximately E8.5 (Fig. 2A; Table 2).

**Table 2:** Developmental ability of *Ets2* mutant embryos.

Parents genotype		Age	No. of embryos	Genotype		
Female	Male			− */* +	+ */em1*	*em1/em1*
*Ets2*^+*/em1*^	*Ets2*^+*/em1*^	E7.5	58	11	26	20
		E8.5	51	14	28	9
		E9.5	14	3	8	3
		E10.5	16	5	8	3

As the frame-shift mutation in *Ets2*^*em1/em1*^ is located in exon 8, the stop codon occurs before exon 9, and the original stop codon is located in exon 10. For this reason, NMD might occur, such that the *em1* mutant mRNA may be degraded in *Ets2*^*em1/em1*^ embryos. To confirm this, we performed RT-PCR using E7.5 embryos. Embryos were separated into the posterior trophectoderm (TE) and anterior epiblast (Epi). Both regions expressed *em1* mutant mRNA (Fig. 2B, and full-length gels are presented in Supplemental Fig S2), and their sequences included 205 nt deletions that were predicted from the genomic sequence. This result showed that em1 mutant RNA had escaped NMD. Furthermore, it is likely the case that the *Ets2*^*em1/em1*^ exhibited a distinct phenotype compared with *Ets2*^*db1/db1*^ even if the *em1* mRNA was translated into a protein product (Supplemental Fig. S7).

### Establishment of *Ets2* homozygous mutant ESC lines and phenotypic analysis after birth

By rescuing placental function using the tetraploid complementation method, *Ets2*^*db1/db1*^ offspring were successfully developed to term. Therefore, we attempted the same experiment to define whether the embryonic lethal phenotype of *Ets2*^*em1/em1*^ was dependent on the placental deficiency or not.

Before conducting tetraploid complementation, we established *Ets2*^*em1/em1*^ and *Ets2*^*em2/em2*^ ESC lines. In this way, we improved the efficiency of obtaining homozygous mutant mice because the ratio of homozygous mutant embryos was only one out of four when we used embryos from a heterozygous crossing. After crossing heterozygotes, two-cell embryos were collected and developed until the blastocyst stage. ESC lines were established from the collected blastocysts and analyzed by genotyping. The rate of homozygous mutant ESC line establishment for both the *Ets2*^*em1/em1*^ and *Ets2*^*em2/em2*^ mutants followed Mendel’s law (Table 3).

**Table 3:** Establishment of *Ets2*^*Δ/Δ*^ ESC lines from blastocysts.

Parents genotype		No. of blastocyst	No. of established ESC lines	Genotype (*)		
Female	Male			+ */* +	+ */Δ*	*Δ/Δ*
*Ets2*^+*/em1*^	*Ets2*^+*/em1*^	56	43	15	18	10
*Ets2*^+*/em2*^	*Ets2*^+*/em2*^	34	21	2	10	8

(*) em1 or em2 allele shown as "Δ".

**Figure 3:**
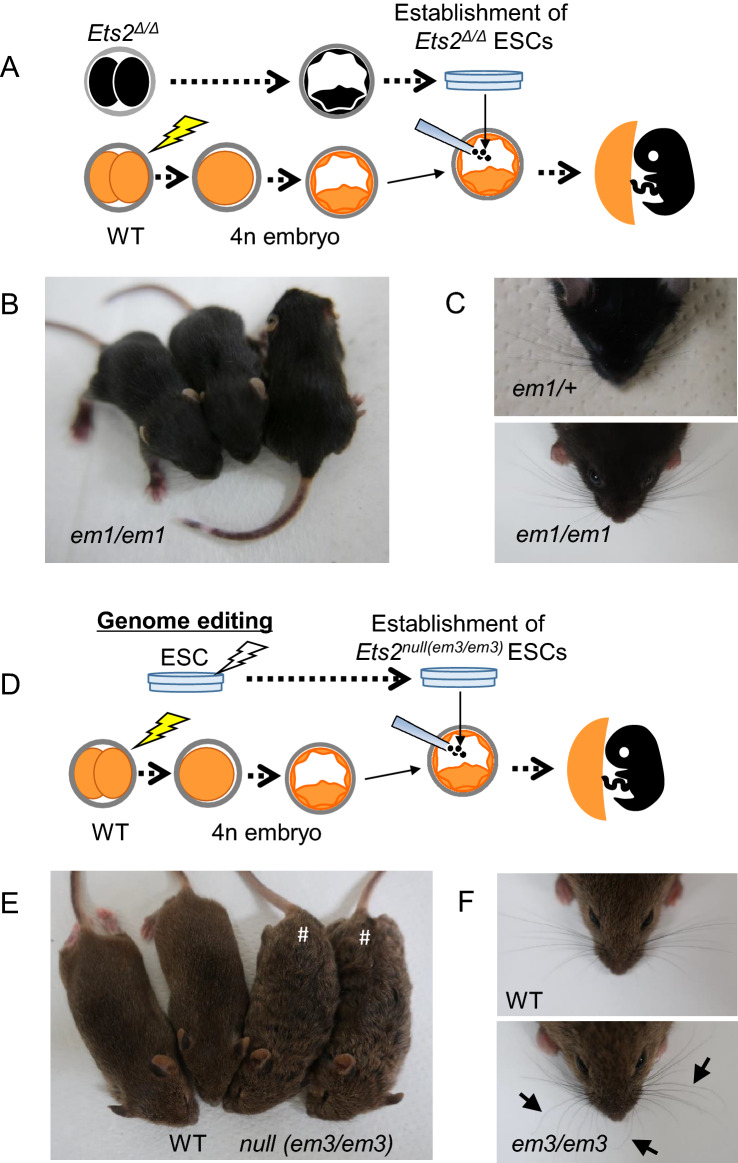
Generation of homozygous *Ets2* mutant models using ESCs and assessment of hair and whisker phenotypes after birth. **(A)** Strategy for the generation of *Ets2* homozygous mutant mouse after establishing the ESC from homozygous mutant blastocysts with tetraploid complementation. **(B)** 2-week-old *Ets2*^*em1/em1*^ mice. **(C)** Faces of 4-week-old in *Ets2*^+*/em1*^ (left picture) and *Ets2*^*em1/em1*^ (right picture). Curly whiskers were not observed in *Ets2*^*em1/em1*^. **(D)** Strategy for the generation of *Ets2* homozygous null mutant mouse after genome-editing to the ESC with tetraploid complementation. **(E)** The hair of the *Ets2*^*null(em3/em3)*^ mutant mouse was wavy (indicated as #). **(F)** Some whiskers of the null mutant mouse were not straight (arrow in the lower picture).

Using the tetraploid complementation method, offspring were obtained from *Ets2*^*em1/em1*^ and *Ets2*^*em2/em2*^ ESC lines (Fig. 3A–C; Supplemental Fig. S3, and Table 4). This result indicated that the embryonic lethality observed for the *Ets2*^*em1/em1*^ and *Ets2*^*em2/em2*^ genotypes was due to a dysfunction of placental differentiation, the same as that seen for the *Ets2*^*db1/db1*^ mutant. Unexpectedly, wavy hair and curly whisker phenotypes were not observed in *Ets2*^*em1/em1*^ or *Ets2*^*em2/em2*^ mice (Fig. 3B,C; Supplemental Fig. S3).

**Table 4:** 4n complementation of *Ets2* homozygous mutant ESC lines.

Genotype	ESC lines	No. of transferred	No. of offspring	No. of wean	Hair phenotype	
					Normal	Wavy
*Ets2*^*em1/em1*^	1A1	40	5	2	2	0
	1A3	20	3	2	2	0
	1B5	78	8	2	2	0
*Ets2*^*em2/em2*^	#11	20	4	2	2	0
	#22	20	6	3	3	0
*Ets2 *^*null (em3/em3)*^	2-10G	56	17	17	0	17
*Ets2 WT*	mF1-05	57	8	8	8	0

To corroborate the relationship between Ets2 and the wavy hair phenotype, we established a null mutant (hereafter referred to as *Ets2*^*null(em3/em3)*^) ES cell line, in which a region upstream of exon 2 through the 3′-UTR of exon 10 was deleted, including all open reading frame (ORF) regions (Supplemental Fig. S4). Pups were then generated using the tetraploid complementation method (Fig. 3D–F; Table 4). Both the wavy hair and curly whisker phenotypes were observed in *Ets2*^*null(em3/em3)*^ mice from around 2-weeks of age, as was observed for *Ets2*^*db1/db1*^ mice.

### Gene expression in newly established *Ets2* mutant skins

In this study, newly established *Ets2*^*em1/em1*^ and *Ets2*^*em2/em2*^ mice exhibited an embryonic lethal phenotype due to placental dysfunction but did not exhibit the wavy hair phenotype of *Ets2*^*db1/db1*^ and *Ets2*^*null(em3/em3)*^ mice, despite having a frame-shift mutation. Therefore, we next investigated the *Ets2* gene expression from the *Ets2* locus in their skins.

**Figure 4:**
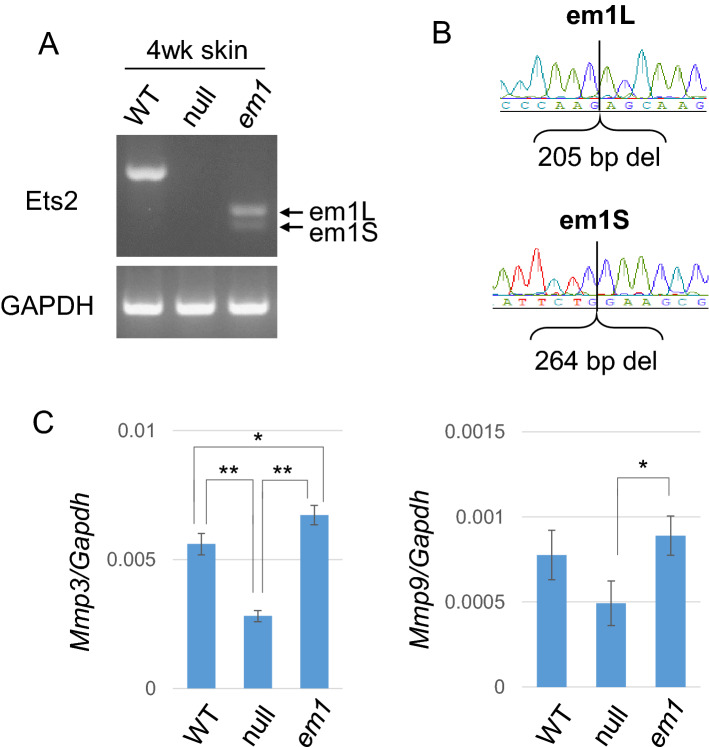
Gene expression in the *Ets2*^*em1/em1*^ skin. **(A)** Gene expression from the *Ets2* locus in skins of WT, *Ets2*^*null(em3/em3)*^ mutant (null) and *Ets2*^*em1/em1*^ (em1) mice. From em1 skin, two types of mRNA were expressed, although both were shorter than WT. The larger mRNA was called em1L, and the smaller mRNA was called em1S. (**B)** Sequences of em1L and em1S. Em1L was the expected sequence, but em1S contained a deleted locus that matched exon 8, shown in Supplemental Fig. S5. **(C)** Gene expression of *Mmp3* and *Mmp9* in the *Ets2*^*em1/em1*^ skin. There were significant differences in the expression level of *Mmp3* between WT and null, em1 and null, and WT and em1. The expression level of *Mmp9* in the null skin was slightly decreased compared with WT, but not significantly, and em1 showed a significant difference compared with null. *p < 0.05, **p < 0.01.

The expression of the mRNA was detected in the skin of wildtype, *Ets2*^*em1/em1*^ and *Ets2*^*em2/em2*^ mice, but not in the skin of *Ets2*^*null(em3/em3)*^ mice. Notably, two sizes of fragments were detected in *Ets2*^*em1/em1*^ and *Ets2*^*em2/em2*^ skin samples (Fig. 4A, Supplemental Fig. S5A and full-length gels are presented in Supplemental Fig. S6).

We hypothesized that the unexpected fragment size might be attributed to a splice variant and that this could explain the differences in phenotype between new *Ets2* mutants and *Ets2*^*db1/db1*^ animals. Therefore, we next analyzed the sequences of the potential splice variants. Although the large bands observed for skins and thymuses of the *Ets2*^*em1/em1*^ and *Ets2*^*em2/em2*^ represented the expected frame-shifted sequences, the sequences of the smaller bands showed skipping of exon 8, which was in-frame and consisted of 264 bp (Fig. 4B; Supplemental Fig. S5B).

Further, we examined the gene expression of *MMP-3* and *MMP-9* in *Ets2*^*em1/em1*^ skins, since a previous report showed that expression of these genes was decreased in *Ets2*^*db1/db1*^ mice^4^. However, the expression levels of *MMP-3* and *MMP-9* in 4-week-old *Ets2*^*em1/em1*^ skins were not reduced compared with the wildtype, even though they were reduced in the *Ets2*^*null(em3/em3)*^ (Fig. 4C).

### Presence of Ets2 mutant protein in the skin and thymus

This Ets2 mutant protein, which skipped exon 8 (Ets2 ΔEx8), was predicted to contain the ETS domain based on the SMART online database^14^ (Supplemental Fig. S7). To confirm the presence of Ets2 mutant protein in the skin and thymus, we performed western blotting. Amino acid sequence sizes of Ets2, Ets2 em1, Ets2 em2, Ets2 ΔEx8 protein were of 468AA, 342AA, 298AA, and 380AA, respectively. Ets2 protein and Ets2 mutant proteins were distinguished by these molecular weights and the lack of band in the null mutant tissues.

**Figure 5:**
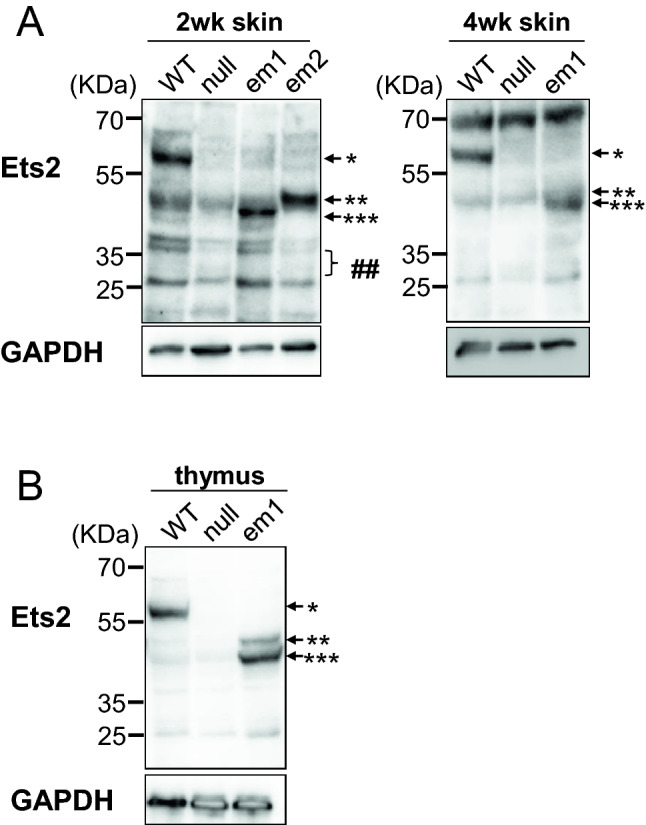
Presence of proteins in the *Ets2* mutant skin and thymus. Western blotting with anti-Ets2 antibody and anti-Gapdh antibody in the skin **(A)** and thymus **(B)**. Native Ets2 protein (*) was detected only in WT samples. Ets2 ΔEx8 (**) and Ets2 em1 (***) mutant proteins were not detected in WT and null mutant samples. Ets2 em2 mutant protein (the predicted size indicates as ##) were not detected in *Ets2*^*em2/em2*^ (em2) skin.

The Ets2 em1 protein was detected at its expected size in *Ets2*^*em1/em1*^ skin and thymus. The Ets2 em2 protein was not found in both *Ets2*^*em2/em2*^ skin, even though Ets2 em2 mRNA (em2-L) was detected in the *Ets2*^*em2/em2*^ skin (Supplemental Fig. S5A and Supplemental Fig. S6B). Ets2 ΔEx8 protein, which has ETS domain, was present in the *Ets2*^*em1/em1*^ thymus, *Ets2*^*em2/em2*^ skin, but not clearly in the *Ets2*^*em1/em1*^ skin (Fig. 5, and full-length blots of Fig. 5 are presented in Supplemental Fig. S8).

## Discussion

In this work, we attempted to generate *Ets2* homozygous mutant models at F0 generation by combination with CRISPR/Cas9 system and tetraploid complementation. We could not obtain the *Ets2* homozygous mutant model at F0 generation using zygotes but generated those using ES cells. In the previous study, the theoretical birth rate of *Ets2*^*db1/db1*^, which crossed with heterozygous parent and performed tetraploid complementation, was guessed 25% along with Mendelian law. However, it has been reported that the actual birth rate was 10%^4^. In this study, the birth rate of offspring from *Ets2*^*null(em3/em3)*^ ES cells by tetraploid complementation was comparable to that from wild type ES cells as shown in Table 4. These results suggest that the mutant ES cells can be used to analyze gene functions after birth if the mutations were deleterious to the trophectoderm lineages.

Here, we newly generated two *Ets2* frame-shift mutant models, namely, *em1* and *em2*. Since both have the stop codon located before the ETS domain, we predicted that both mutant proteins would lack the ETS domain if they were translated. Therefore, we expected that the phenotypes in these homozygous mutants would mimic those described in previous reports for *Ets2*^*db1/db1*^ mice. Indeed, *Ets2*^*em1/em1*^ mice exhibited the same embryonic lethal phenotype, but the wavy hair and curly whisker phenotypes observed in *Ets2*^*db1/db1*^ and *Ets2*^*null(em3/em3)*^ mice did not occur in *Ets2*^*em1/em1*^ and *Ets2*^*em2/em2*^ mice. Hair and whisker phenotypes of *Ets2*^*db1/db1*^ mice have been caused due to the lack of ETS domain^4^. We found that the skin and thymus of *Ets2*^*em1/em1*^ and *Ets2*^*em2/em2*^mice expressed a mutant mRNA lacking exon 8 that could potentially be translated into a protein, including the ETS domain. We detected Ets2 ΔEx8 protein in *Ets2*^*em2/em2*^ skin and *Ets2*^*em1/em1*^ and *Ets2*^*em2/em2*^ thymuses. These data demonstrated that Ets2 ΔEx8 mRNA was translated into the protein which contains the ETS domain. Therefore, we hypothesize that this Ets2 ΔEx8 protein could rescue the Ets2 function in hair and whisker. This was strongly suggested by the finding that these mice exhibited comparable levels of *MMP-3* and *MMP-9* mRNAs to wildtype, and *Ets2*^*db1/db1*^ and *Ets2*^*null(em3/em3)*^ mice exhibited decreased expression.

DNA mutations in a genetic locus frequently lead to exon skipping, and several human diseases are linked to these types of exon skipping events. In addition, exon skipping can occur because of lack of exonic splicing enhancer sequences or an exonic splicing silencer sequence inside of an exon^15,16^. Some reports have also suggested that unexpected exon skips can occur when using the CRISPR/Cas9 system, indicating that a frame-shifted mutant exon induced by CRISPR/Cas9 is skipped but can be induced through alternative splicing or in-frame exon skipping^17–20^. These studies indicated that mutations located on the three multiple exons could induce its exon skipping. Due to our targeted exon 8 in *Ets2* gene consists of 264 nts (88 multiples of three nts), exon skipping may have occurred in the tissue specifically.

Since there was no report about the mutation within exon 8 of Ets2 in human by ClinVar database^21^, the new *Ets2* frame-shift mutant models could not applied to the human disease model. However, these new *Ets2* frame-shift mutant models had the following issue.

The exon skip observed was only identified in the skin and thymus but not detected during embryonic stages. Thus, the induction of the exon skip was likely due to the changes in mRNA splicing that were dependent on the cell type or developmental stage, despite having the same genomic mutation. This phenomenon has a possible effect on the appearance of phenotypes making it a novel finding of this study.

Overall, our study suggested that all ORF-deletion models are adequate for analysis if we have to generate the gene knockouts. Furthermore, research related to indel mutation or point mutation in human disease models need to pay attention to the effects of alternative splicing.

## Methods

### Animals

All animal experiments were conducted in accordance with the guidelines of “Regulations and By-Laws of Animal Experimentation at the Nara Institute for Science and Technology”, and were approved by the Animal experimental Committee at the Nara Institute of Science and Technology (the approval no. 1639). Study of the animal experiments were carried out in compliance with the ARRIVE guidelines^22^. B6D2F1 female mice and ICR mice were purchased from SLC (Japan). C57BL/6J male mice were purchased from CLEA (Japan).

### Collection of zygotes

Female mice were treated by PMSG and hCG for superovulation, then mated with male mice. Pronuclear stage zygotes were collected from female oviducts after 20 h of hCG injection. After removing cumulus cells using hyaluronidase, zygotes were incubated in KSOM at 37 °C under 5% CO2 in the air until use. 2-cell stage zygotes were collected from female oviducts after 42–46 h of hCG injection by the flush-out method. Collected 2-cell stage embryos were incubated until use the same as above.

### Generation of *Ets2* mutant zygote by CRISPR/Cas9 system using electroporation

Target sites of guide RNA (gRNA) were designed using the web tool CRISPR direct^23^. Genome editing by the electroporation was referred to as the previous study^12^.

CFB16-HB and LF501PT1-10 electrode (BEXCo.Ltd., Tokyo, Japan) were used for electroporation. 30–40 pronuclear stage zygotes were subjected to electroporation at one time. Zygotes were washed with Opti-MEM I (Thermofisher) three times, subsequently placed in a line in the electrode gap filled with five μl the mixture of 120 ng/μl Cas9 protein (TaKaRa, Japan), 300 ng/μl *tracrRNA,* and 200 ng/μl *crRNA* (HPLC grade, Fasmac) in Opti-MEM I. The electroporation condition was performed were 30 V (3 ms ON ± 97 ms OFF) four times. After electroporation, zygotes were washed with KSOM three times then cultured until developing the eight-cell stage. Eight-cell stage embryos were provided to the tetraploid complementation.

### Establishment of *Ets2* mutated ESC lines

To establish the* Ets2* homozygous mutant model, collected 2-cell stage embryos from *Ets2* heterozygous mutant parents were incubated until the blastocyst stage, removing the Zona pellucida (ZP) using Acidic Tyrode solution (Sigma T1788). Blastocyst embryos without the ZP were seed on gelatin-coated 60-mm dishes and cultured on mouse embryonic fibroblast (MEF) with N2B27 medium supplemented with 3 μM CHIR99021(Axon1386), 1.5 μM CGP77675 (Sigma SML0314), and mouse LIF (N2B27-a2i/L medium)^24^. After seven days, the outgrowth of blastocysts was disaggregated by 0.25% trypsin in 1 mM EDTA in PBS (−). Half of the cells were seeded on MEF with the gelatin-coated dishes for expanding. The others were seeded on the gelatin-coated dishes without MEF for genotyping by PCR. *Ets2* homozygous mutant ESC lines were provided for tetraploid complementation.

The *Ets2* null mutant model was established using ESCs, referred to as the previous study^25^. mF1-05 ESC line, which was newly established from 129X1and C57BL6/J F1 embryo, was seeded on MEF then transfected with two designed pSpCas9(BB)-2A-Puro (pX459) V2.0 (Addgene #62988) plasmids using Lipofectamine 3000 (Thermofisher). Transfected cells were selected by transient treatment with 1 μg/ml puromycin, then ESC colonies were subject to genotyping with PCR and sequencing. The *Ets2* null mutant ESC line was provided for tetraploid complementation.

### Tetraploid complementation

Tetraploid embryos were prepared as described previously^9,26^. In brief, ICR two-cell stage embryos were placed in the fusion buffer, and electrofusion was performed by applying 140 V for 50 ms after aligning embryos between the electrodes. CFB16-HB and LF501PT1-10 electrode (BEXCo.Ltd., Tokyo, Japan) were used for cell fusion.

A wild-type tetraploid four-cell embryo and a genome-edited diploid eight-cell embryo were aggregated after removing the Zonae pellucid for the aggregation method. For the injection method, *Ets2* mutant ESCs were injected into a wild-type tetraploid four-cell embryo or blastocyst. These embryos were cultured until the blastocysts stage and transferred into the uterus of E2.5 pseudopregnant ICR mice. Offspring were recovered by natural delivery or Caesarean section on E19.5. The mutation of offspring was detected by genotyping with PCR and sequencing.

### Genotyping

Genotyping primers for detecting *Ets2*-wild, em 1, and em 2 alleles were 5′-ctgagtttaagagtgctcggagg-3′ (Ets2_Fw) and 5′- gccctataggacttgtgtacagg-3′ (Ets2_Rev). Primers for Ets2 null (em3) mutant allele(s) were 5′-tgtggagtctcacatcgaag-3′ (Ets2_Ex2_F) and 5′- gggcctgctcggtgccacgg-3′ (Ets2_EX10_R). DNA fragments were amplified using GoTaq (Promega) for 40 cycles under the following conditions: 94 °C for 30 s, 60 °C for 30 s and 68 °C for 40 s for detecting wild, em1 or em2 allele, and 94 °C for 30 s, 60 °C for 30 s and 68 °C for 20 s for detecting the null allele, respectively.

### RNA expression analysis

Mouse cDNAs were prepared from 4-week old skin, adult skin, and adult thymus using SuperScript III Reverse Transcriptase (Thermo Fisher Scientific) after purified RNA by Trizol reagent (Thermo Fisher Scientific). RT-PCR was performed using 20 ng of cDNA with the following primers: 5′-CGTGAATTTGCTCAACAACAATTCTG-3′ and 5′-GAGAGGCTATGCCGGT-3′ for *Ets2*, 5′-CCAGTATGACTCCACTCACG-3′ and 5′-GACTCCACGACATACTCAGC-3 for *Gapdh* ^27^. cDNA fragments were amplified using KOD Fx Neo (TOYOBO) or GoTaq (Promega) for 35 cycles under the following conditions: 94 °C for 30 s, 60 °C for 30 s and 72 °C for 40 s for *Ets2*, and 94 °C for 30 s, 53 °C for 30 s and 72 °C for 30 s for *Gapdh*.

Quantitative Real-time PCR was performed using 20 ng of cDNA with following primers: 5′-TTAAAGACAGGCACTTTTGG-3′ and 5′-CAGGGTGTGAATGCTTTTAG-3′for *Mmp3*, 5′-CGTCTGAGAATTGAATCAGC-3′ and 5′-AGTAGGGGCAACTGAATACC-3′ for *Mmp9* expression^5^. Gene expression level was normalized by Gapdh, the same cDNA. The primer set for *Gapdh* was the same as above. Real-time PCR was performed by LightCycler96 (Roche) using the Luna Universal qPCR Master Mix (NEB), and the data were analyzed by the LightCycler96 software (Roche, version 1.1.0.1320).

### Western blotting

Whole cell extracts of 2 weeks old skin or thymus were prepared by homogenization in RIPA buffer (Nacalai). Equivalent amount of protein were separated by 4–12% Bolt Bis–Tris gel (Invitrogen) and run under reducing conditions. After gel electrophoresis, they transferred to immobilon-P membrane (Merck). Membranes were blocked in 5% skim milk powder in phosphate-buffered saline with Tween20. The membrane was incubated with anti-Gapdh (WAKO, 010-25521) or anti-Ets2 (Genetex, GTX104527) followed by a secondary antibody conjugated with horseradish peroxidase (Cytiva, NA931, NA934). Peroxidase activity was detected with Chemi-Lumi One (Nacalai) using the FUSION -Chemiluminescence Imaging System (Vilber-Lourmat).

### Statistics analysis

The statistical difference was determined using the Student t-test. Differences were considered statistically significant if the P-value was less than 0.05.

## Supplementary Information

Supplementary Figures.
