# Supplementary material for: A novel tissue specific alternative splicing variant mitigates phenotypes in Ets2 frame-shift mutant models
Source: Sci Rep. 2021 Apr 15;11:8297. doi: 10.1038/s41598-021-87751-5 (PMC8050053; doi:10.1038/s41598-021-87751-5)

Supplementary information

Title: A novel tissue specific alternative splicing variant mitigates phenotypes in *Ets2* frame-shift mutant models

Yuki Kishimoto [1#], Iori Nishiura [1#], Wataru Hirata [1#], Shunsuke Yuri [1], Nami Yamamoto [1], Masahito Ikawa [2], and Ayako Isotani [1*]

[1] Division of Biological Science, Graduate School of Science and Technology, Nara Institute of Science and Technology, 8916-5 Takayama-cho, Ikoma, Nara, 630-0192, Japan

[2] Research Institute for Microbial Diseases, Osaka University, 3-1 Yamadaoka, Suita, Osaka 565-0871, Japan

* Corresponding author

Ayako Isotani

E-mail address: isotani@bs.naist.jp

# Equal contribution of authors

Supplemental Fig. S1 The full-length gel data of Fig. 1C.

Supplemental Fig. S2 The full-length gels data of Fig. 2B.

Supplemental Fig. S3 Hair phenotype of *Ets2*$^{em2/em2}$model

Supplemental Fig. S4 Generation of the Ets2 null mutant mouse. A, Design of sgRNA targeting sites and checking primer positions. B, Genotyping of *Ets2*$^{null(em3/em3)}$ mutant mouse. The Ets2 null mutant allele was detected by the Ex2-F and Ex10-R primer set but not detected by the Ets2-F and Ets2-R primer set.

Supplemental Fig. S5 cDNA sequence from the Ets2 region. A, RT-PCR of skin and thymus cDNA samples. *Ets2*$^{em1/em1}$ (em1) thymus expressed two mutant mRNA, the same as that observed for the skin, shown in Fig. 4A. *Ets2*$^{em2/em2}$ (em2) skin and thymus also expressed two mutant mRNAs, namely, em2-L and em2-S. The band size of em2-S was the same as that of em1-S. B, Sequences of the deleted region in em2-L and em2-S, respectively. C, Sequence information for Ets2 mutant cDNAs. The em1L and em2L sequences were the same as those predicted from genomic mutations. The em1S and em2S sequences were the same and matched the exon 8-skipped sequence.

Supplemental Fig. S6 A, The full-length gels data of Fig. 4A. B, The full-length gels data of Supplemental Fig. S5A.

Supplemental Fig. S7 Predicted mutant Ets2 amino acid information from the expressed cDNA sequences. A, Predicted mutant Ets2 amino acid sequences. B Predicted mutant Ets2 protein's domain information.

Supplemental Fig. S8 The full-length blots data of Fig. 5.

Supplemental Fig. S1 Y Kishimoto et al.

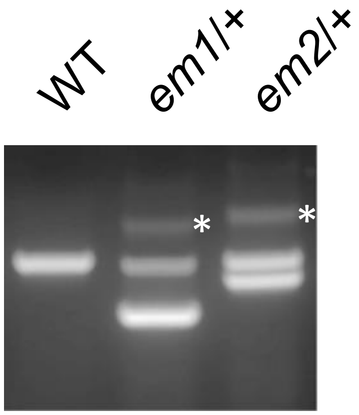

“*” show non-specific bands.

Supplemental Fig. S2 Y Kishimoto et al.

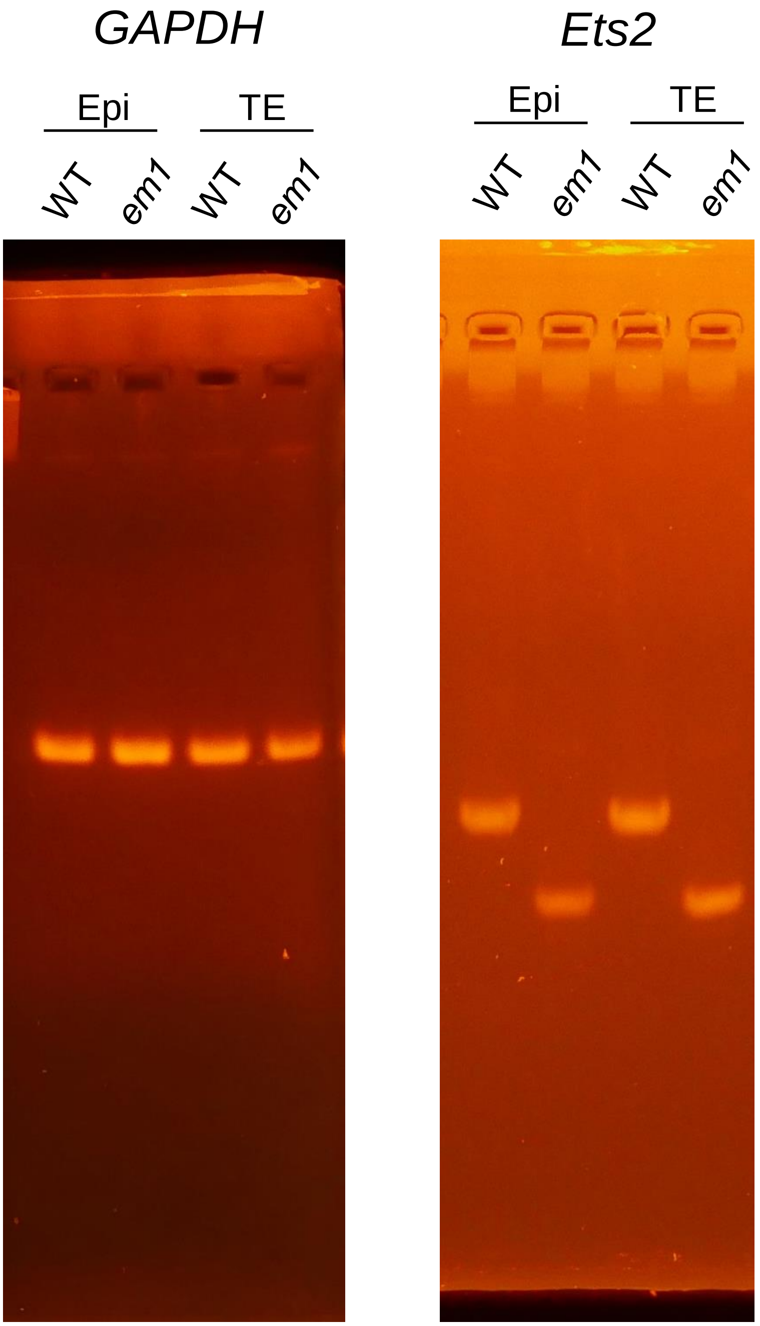

em2/em2

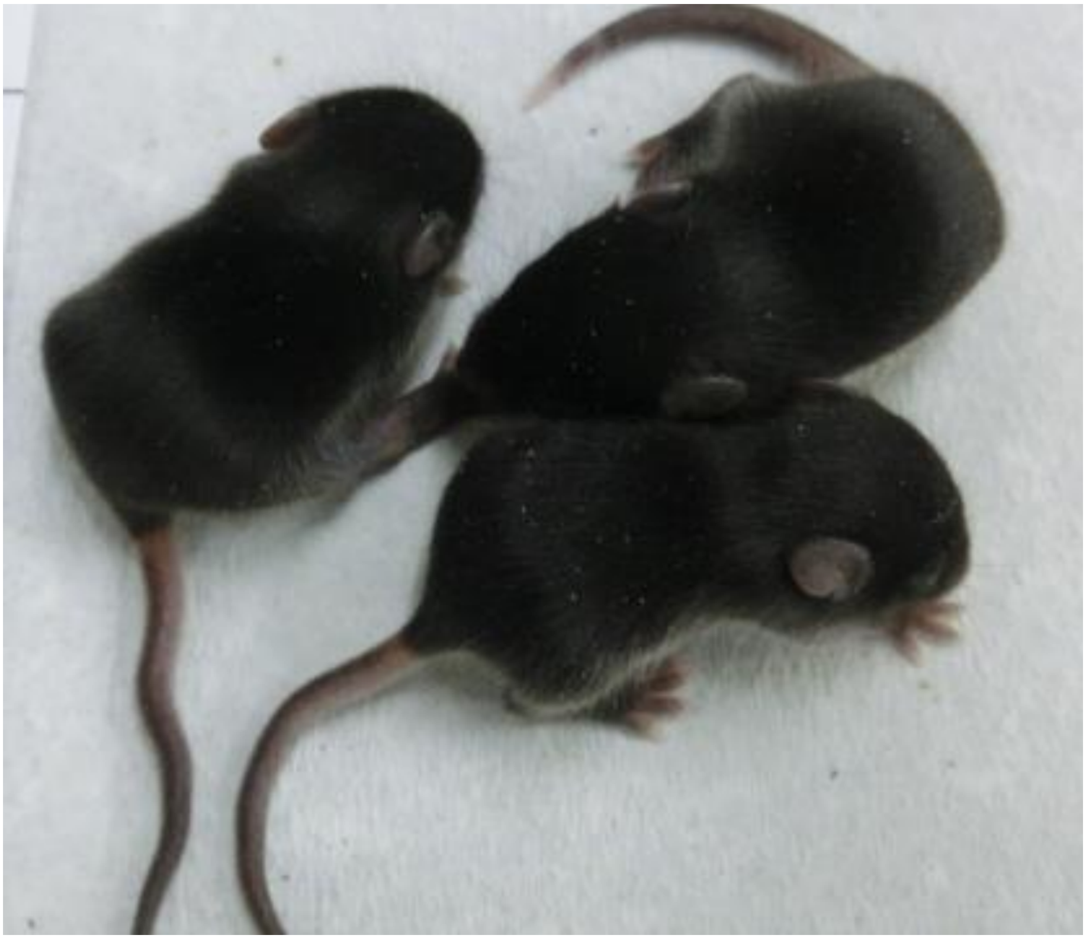

Supplemental Fig. S4 Y Kishimoto et al.

A

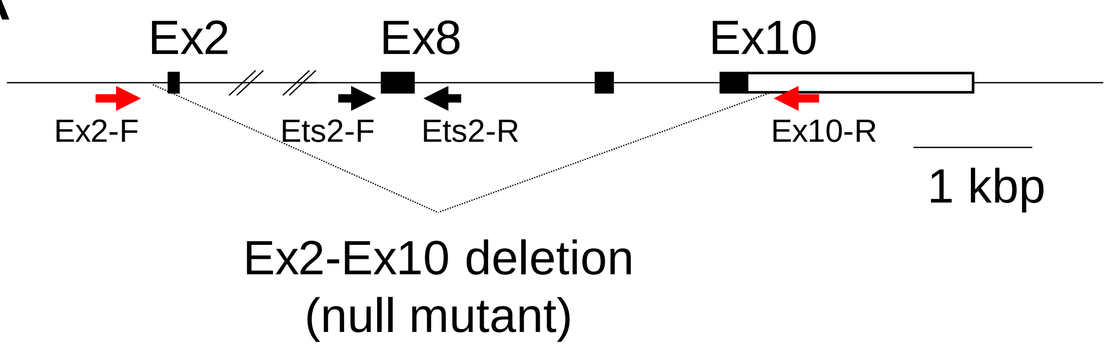

B

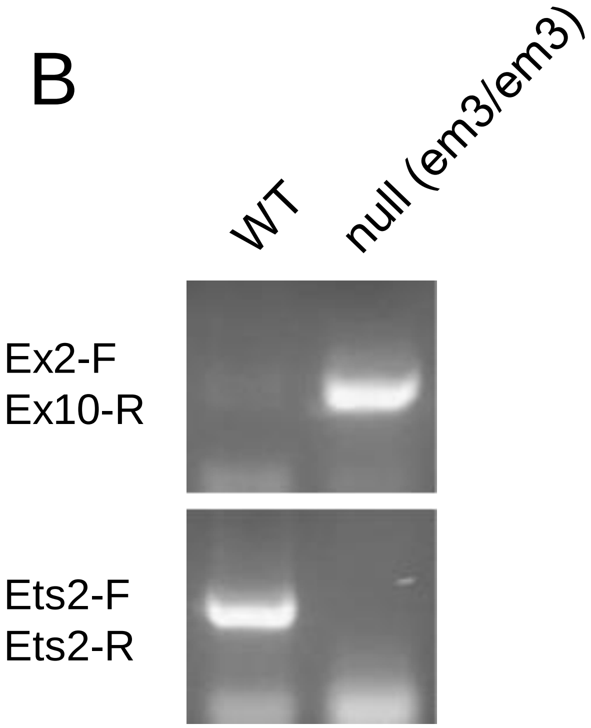

Supplemental Fig. S5 Y Kishimoto et al.

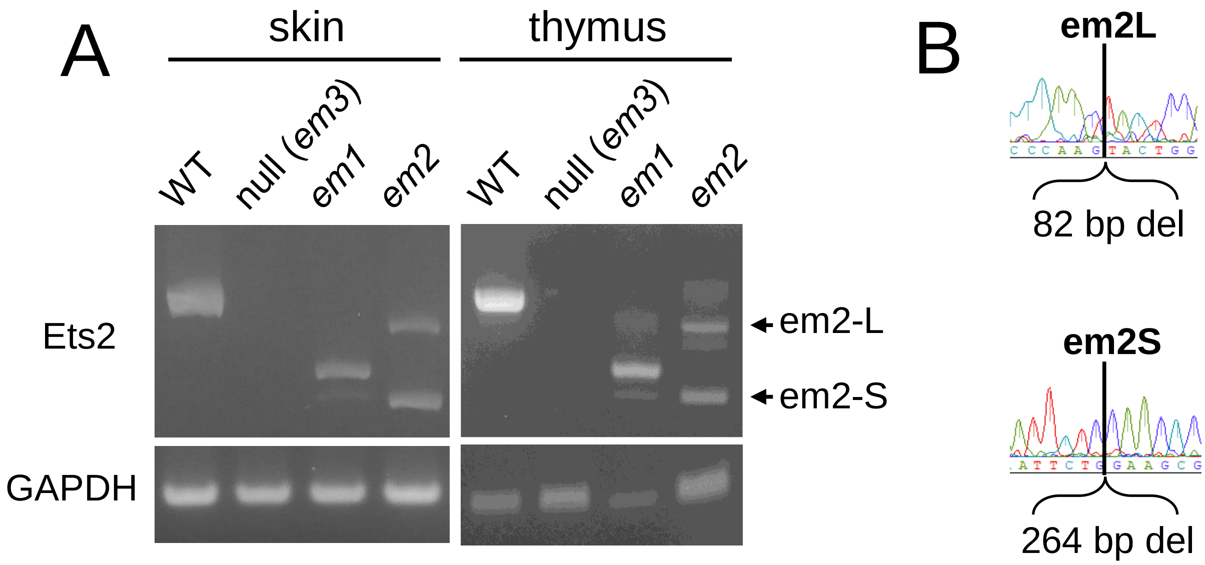

C

```
                              exon7 <-|-> exon8
WT         1 CGTGAATTTGCTCAACAACAATTCTGGAAAACCCAAGGACCACGACTCTC 50
em1L       1 CGTGAATTTGCTCAACAACAATTCTGGAAAACCCAAG------------- 37
em2L       1 CGTGAATTTGCTCAACAACAATTCTGGAAAACCCAAGTACTGGATGTACA 50
em1/2S     1 CGTGAATTTGCTCAACAACAATTCTG------------------------ 26
Ex8skip    1 CGTGAATTTGCTCAACAACAATTCTG------------------------ 26

WT        51 CAGAGAACGGTGGGGACAGCTTCGAGAGCTCCGACTCGCTGCTGAGGTCC 100
em1L      37 -------------------------------------------------- 37
em2L      51 GCGGGTACCTTCCTTCGAGAGCTTTGAGGAGGACTGTAGCCAGTCTCTGT 100
em1/2S    26 -------------------------------------------------- 26
Ex8skip   26 -------------------------------------------------- 26

WT       101 TGGAACAGCCAGTCGTCCCTACTGGATGTACAGCGGGTACCTTCCTTCGA 150
em1L      37 -------------------------------------------------- 37
em2L     101 GCCTCAGTAAGCTGACCATGTCCTTCAAGGACTACATCCAAGAGAGGAGC 150
em1/2S    26 -------------------------------------------------- 26
Ex8skip   26 -------------------------------------------------- 26

WT       151 GAGCTTTGAGGAGGACTGTAGCCAGTCTCTGTGCCTCAGTAAGCTGACCA 200
em1L      37 -------------------------------------------------- 37
em2L     151 GACCCAGTCGAGCAAGGCAAACCAGTTATTCCTGCAGCAGTACTGGCTGG 200
em1/2S    26 -------------------------------------------------- 26
Ex8skip   26 -------------------------------------------------- 26

WT       201 TGTCCTTCAAGGACTACATCCAAGAGAGGAGCGACCCAGTCGAGCAAGGC 250
em1L      38 ------------------------------------------AGCAAGGC 45
em2L     201 CTTCACTGGAAGCGGACCAATCCAGTTGTGGCAGTTTCTTCTGGAGCTAC 250
em1/2S    26 -------------------------------------------------- 26
Ex8skip   26 -------------------------------------------------- 26

WT       251 AAACCAGTTATTCCTGCAGCAGTACTGGCTGGCTTCACTGGAAGCGGACC 300
em1L      46 AAACCAGTTATTCCTGCAGCAGTACTGGCTGGCTTCACTGGAAGCGGACC 95
em2L     251 TCTCTGACAAGTCCTGTCAATCTTTCATCAGCTGGACGGGGGATGGATGG 300
em1/2S    27 ----------------------------------------GAAGCGGACC 36
Ex8skip   27 ----------------------------------------GAAGCGGACC 36
                                          exon8 <-|-> exon9
```

Supplemental Fig. S6 Y Kishimoto et al.

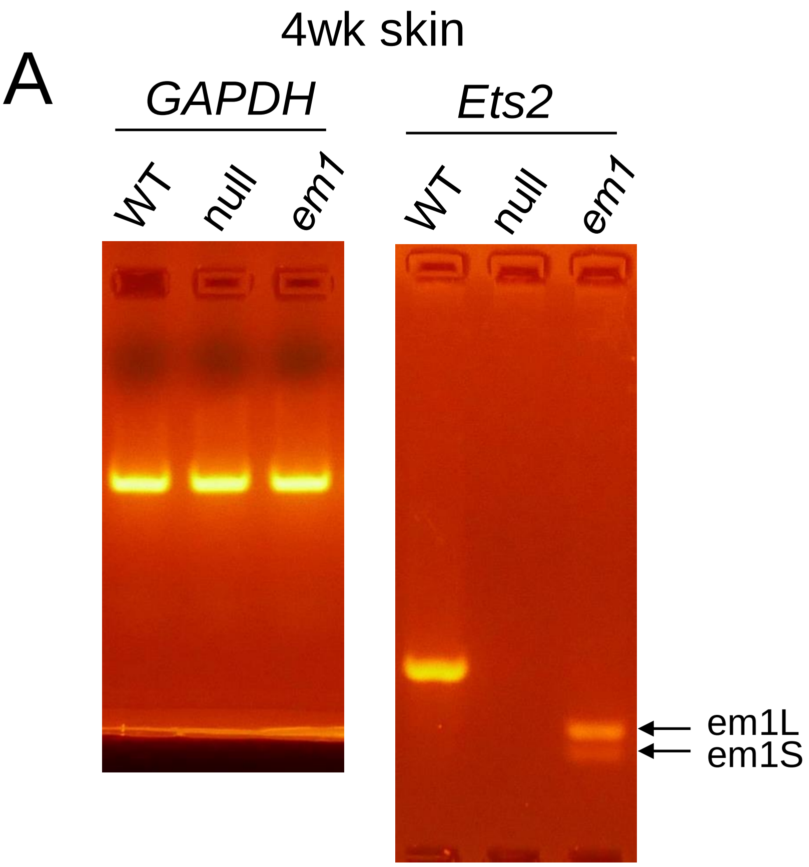

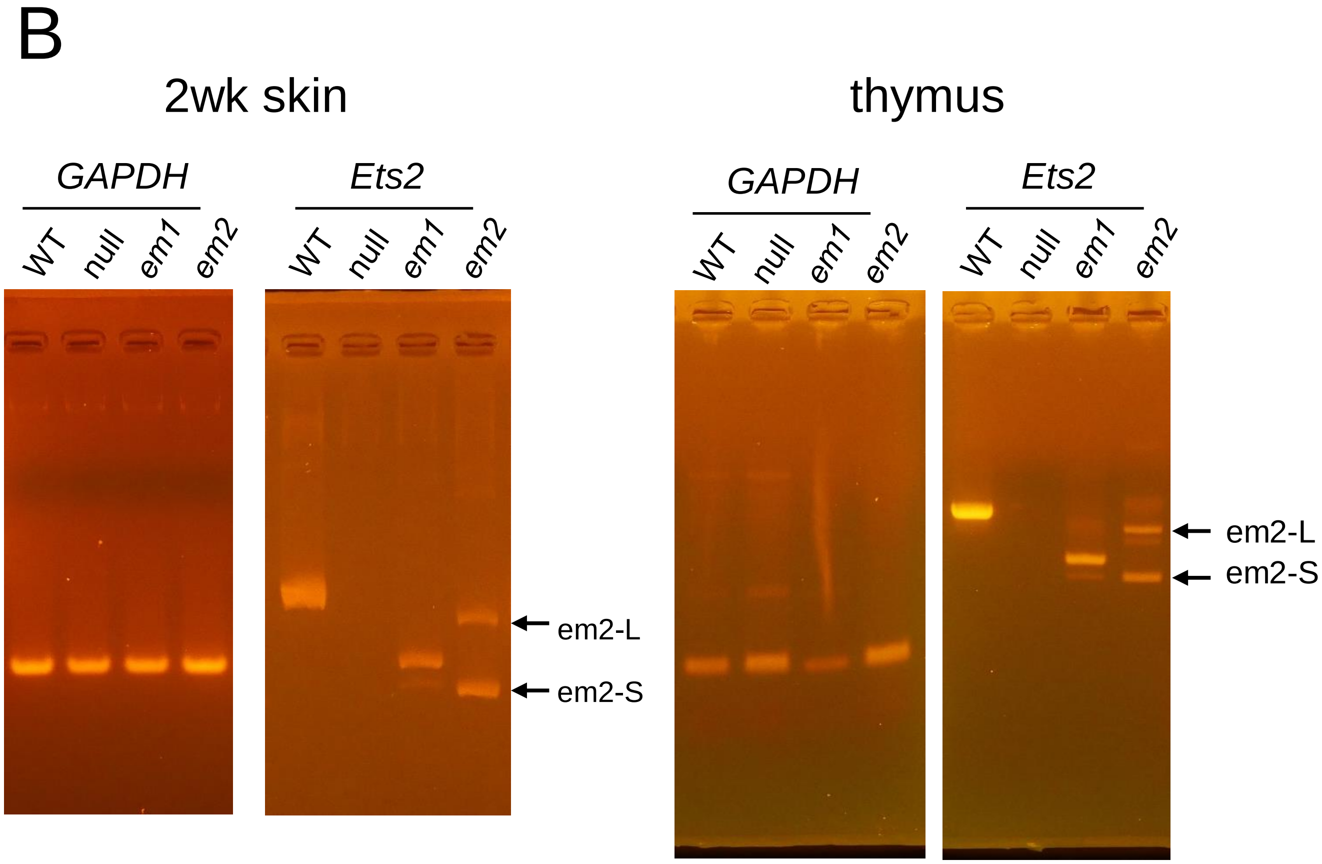

Supplemental Fig. S7 Y Kishimoto et al.

A

```
Ets2NP_035939.3      251 CSISQDFPSSNVNLLNNNSGKPKDHDSPENGGDSFESSDSLLRSWNSQSS 300
Ets2em1_protein      251 CSISQDFPSSNVNLLNNNSGKPKSKANQLFLQQYWLASLEADQSSCGSFF 300
Ets2em2_protein      251 CSISQDFPSSNVNLLNNNSGKPKYWMYSGYLPSRALRRTVASLCASVS-- 298
Ets2Ex8skip_protein  251 CSISQDFPSSNVNLLNNNSG------------------------------ 270
Ets2db1_protein      251 CSISQDFPSSNVNLLNNNSGKPKDHDSPENGGDSFESSDSLLRSWNSQSS 300

Ets2NP_035939.3      301 LLDVQRVPSFESFEEDCSQSLCLSKLTMSFKDYIQERSDPVEQGKPVIPA 350
Ets2em1_protein      301 WSYSLTSPVNLSSAGRGMDGSSSLLTPMRLPAGGGRGKINQR-------- 342
Ets2em2_protein      298 -------------------------------------------------- 298
Ets2Ex8skip_protein  270 -------------------------------------------------- 270
Ets2db1_protein      301 LLDVQRV------------------------------------------- 307

Ets2NP_035939.3      351 AVLAGFTGSGPIQLWQFLLELLSDKSCQSFISWTGDGWEFKLADPDEVAR 400
Ets2em1_protein      342 -------------------------------------------------- 342
Ets2em2_protein      298 -------------------------------------------------- 298
Ets2Ex8skip_protein  271 --------SGPIQLWQFLLELLSDKSCQSFISWTGDGWEFKLADPDEVAR 312
Ets2db1_protein      307 -------------------------------------------------- 307

Ets2NP_035939.3      401 RWGKRKNKPKMNYEKLSRGLRYYYDKNIIHKTSGKRYVYRFVCDLQNLLG 450
Ets2em1_protein      342 -------------------------------------------------- 342
Ets2em2_protein      298 -------------------------------------------------- 298
Ets2Ex8skip_protein  313 RWGKRKNKPKMNYEKLSRGLRYYYDKNIIHKTSGKRYVYRFVCDLQNLLG 362
Ets2db1_protein      307 -------------------------------------------------- 307

Ets2NP_035939.3      451 FTPEELHAILGVQPDTED                                 468
Ets2em1_protein      342 ------------------                                 342
Ets2em2_protein      298 ------------------                                 298
Ets2Ex8skip_protein  363 FTPEELHAILGVQPDTED                                 380
Ets2db1_protein      307 ------------------                                 307
```

B

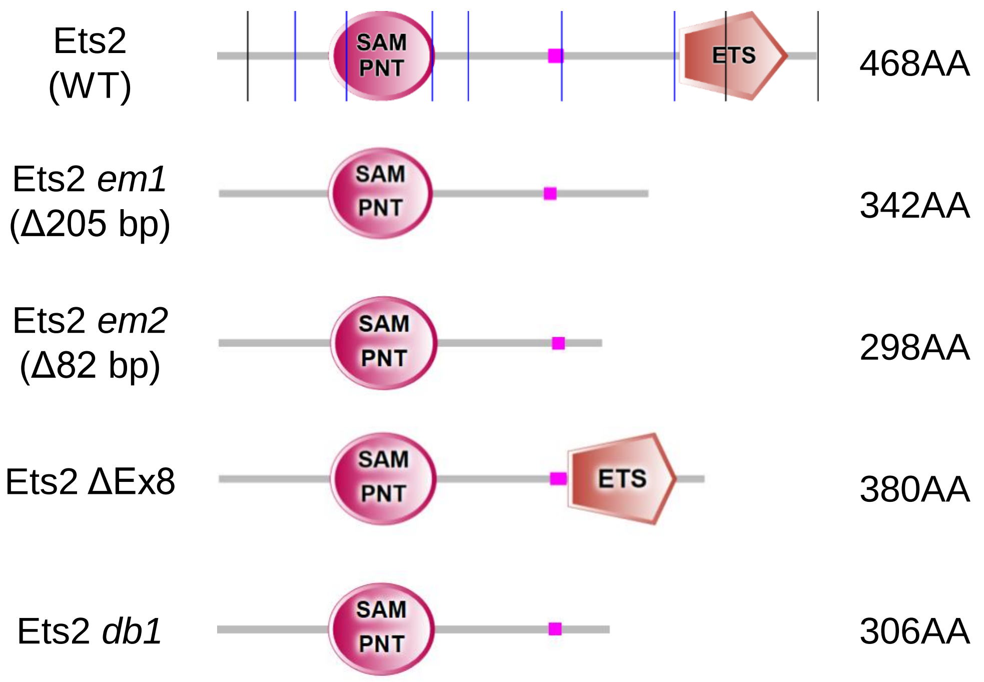

Supplemental Fig. S8 Y Kishimoto et al.

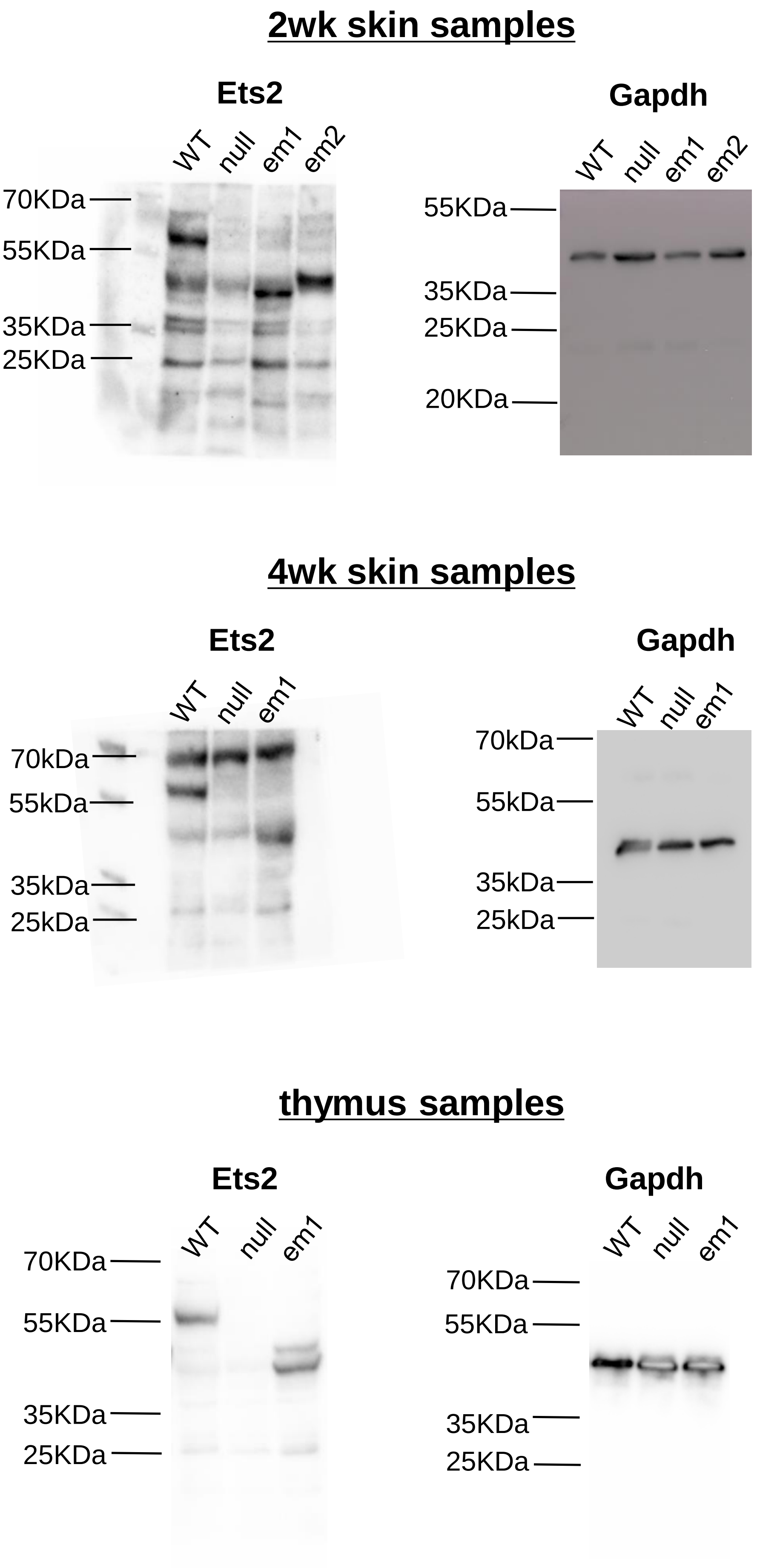

Supplement: Supplementary file 1 — Supplementary Figures. [file 41598_2021_87751_MOESM1_ESM.pdf]
